# Equity Reform and High-Quality Development of State-Owned Enterprises: Evidence From China in the New Era

**DOI:** 10.3389/fpsyg.2022.913672

**Published:** 2022-08-26

**Authors:** Hongwei Liao, Dingqing Wang, Lei Zhu, Jing Zhao

**Affiliations:** ^1^Center for China Public Sector Economy Research, Jilin University, Changchun, China; ^2^School of Economics, Jilin University, Changchun, China; ^3^School of Public Administration, Xi’an University of Architecture and Technology, Xi'an, China; ^4^School of Business Administration, Xi'an Eurasia University, Xi'an, China

**Keywords:** state-owned enterprises, equity reform, high-quality development, inverted U-shaped, China

## Abstract

How to improve the development quality of state-owned enterprises is of great significance to the economic and social development in the transition period. And promoting the reform of mixed ownership is an important path for state-owned enterprises to achieve high-quality development. Based on the micro-data of China’s A-share listed state-owned companies, the paper explores the impact of mixed ownership reform on the high-quality development of state-owned enterprises. It clarifies the importance and moderation of equity reform and the heterogeneity of impact effects from the theoretical mechanism analysis and empirical test. It also analyzes the reasons of inverted U-shape from the perspective of the transmission mechanism of the internal competition atmosphere and non-state-owned capital speculation motivation. It is found that the relationship between equity reform and state-owned enterprises’ high-quality development is inverted U-shaped with multi-dimensional heterogeneity. From the analysis of conduction mechanism, on the one hand, the equity reform can enhance the internal competitive atmosphere, stimulate the vitality of enterprises and improve the development quality for state-owned enterprises. On the other hand, it enhances the speculation motivation of enterprises and slows down the high-quality development process.

## Introduction

The new era is “the new historical orientation of China’s development” since the 18th CPC National Congress (Wang, 2017). As China enters a new era, the focus on scale and speed has given way to quality and efficiency (Hong et al., 2018). Improving the development quality of economic actors has always been an important topic, both in theoretical research and practice (Feng et al., 2021). At present, the research on high-quality development basically focuses on the macro level, leaving a gap to the research on the micro foundation of high-quality economic development for enterprise development. The academic definition of high-quality enterprise development is still blurred. Most scholars claimed that improving production efficiency plays a key role in high-quality economic development of enterprises. They measure the development level of enterprises by the production efficiency and the enterprise performance (Rawski, 1997; Smith, 2014; Molnar, 2017). However, some other scholars suggested that improving total factor productivity is important in the production efficiency and the development quality (Lin, 2015; Cai and Lu, 2018; Cai, 2019). State-owned enterprises are the main body of the national economy, improving the development quality of state-owned enterprises is the key to steady growth and quality improvement. The research group of the Economic Research Institute of the Chinese Academy of Social Sciences (Research Group of Institute of Economics, Chinese Academy of Social Sciences, 2020) pointed out that the state-owned enterprises currently present a pattern of “big but not strong” and “big but not superior” according to its development quality of state-owned capital. At the policy level, the “14th Five-Year Plan” clearly proposed to deepen the reform of state-owned enterprises, making state-owned capital in becoming stronger, doing better and growing bigger, and eventually build world-class enterprises with global competitiveness. The reform of mixed ownership for state-owned enterprises has become an important part of the overall deepening reform in the new era. Promoting a mixed ownership reform of state-owned enterprises, making state-owned capital and state-owned enterprises stronger, better and bigger, and promoting the quality of development of state-owned mixed enterprises is an important step in the reform, and is an important way to ensure the high-quality development of Chinese economy in the new era. Accordingly, high quality development of the state-owned enterprises should have basic attributes of “strong, excellent and big.” And how to improve the competitiveness of state-owned enterprises is a question that needs to be answered.

The equity reform can effectively mitigate the moral hazard and has a positive impact on enterprise development, since there is a principal-agent problem in the process of enterprise operation under the condition of asymmetric information (Ross, 1973). Non-state-owned capital enables state-owned enterprises to have high independent decision-making ability, and thus improves the efficiency (Song et al., 2015; Chen et al., 2021) and the performance levels of the enterprise (Sun and Tong, 2003; Abramov et al., 2017). The mixed oligarch model of win-win cooperation between state-owned capital and non-state-owned capital is beneficial to the development of enterprises (Matsumura, 1998). Anderson et al. (2015) studied the impact of the privatization process of state-owned enterprises on the economic efficiency of enterprises over time and found that the economic efficiency of state-owned mixed enterprises is improved with the increase of long-term non-state ownership. The positive impact of mixed ownership reform on the development of state-owned enterprises has been strongly supported by the existing research. In addition, the effect of the degree of equity reform on the growth of state-owned enterprises has also become a topic of interest recently. The adaptability of mixed reform is important. Certain concentrated and balanced shareholding structures have an obvious motivating and monitoring effect on state-owned capital, which can effectively facilitate the improvement of state-owned enterprise performance and development quality (Bennedsen and Wolfenzon, 2000). Moreover, the equity structure and firm performance show non-simple linear hump-shaped relationship (Coles et al., 2012), while the total factor productivity and development level relationships also show non-simple linear U-shaped relationship (Yin et al., 2018). What is more, Morris et al. (2002) found that the degree of equity reform of state-owned enterprises is constrained by the economic and social reform plan. Although the equity reform of state-owned enterprises is considered to improve the competitiveness and development level, the degree of equity reform is also affected by factors such as the degree of improvement in the economic system (Ji et al., 2005), the degree of government intervention (Su and He, 2012) and the political objectives for developing state-owned enterprises (Kira and Silke, 2014).

The extant literature focuses on whether the state-owned enterprises should carry out the mixed ownership reform, the effect of the mixed ownership reform and the influence of the degree of equity reform on the development of enterprises. Due to varying factors such as the basic situation in a country, stage of development, political objectives and the characteristics of different types of capital, the impact of the equity reform for state-owned enterprises the quality of development will be different in different countries. It is unknown to what extent equity reform can influence state-owned enterprises in China during the period of the establishment and improvement of the socialist economic system. The present research uses the relevant data of state-owned enterprises listed in the Chinese A-share market since the 18th CPC National Congress to explore the specific impact of equity reform on the high-quality development of enterprises in the context of the new era on the basis of heterogeneity analysis. We aim to probe into the effect of the equity reform of state-owned enterprises on the development quality of enterprises from the perspectives of heterogeneity and transmission mechanism, which provides an empirical basis for the policy formulation in the new era.

The marginal contribution of the paper has three main aspects. First, there are few studies on the construction of high-quality development indicators of state-owned enterprises. We construct a high-quality development index of state-owned enterprises based on the standard of “becoming stronger, doing better, and growing bigger,” and explore the impact of equity reform on the high-quality development of state-owned enterprises in the new era. And We found the inverted U-shaped relationship between equity reform and high-quality development of state-owned enterprises. Second, in the process of empirical analysis, based on the principle of “three causes, three preferences, and three disincentives,”^1^ we empirically test the specific impact of equity reform on high-quality development of state-owned enterprises from the time dimension and the perspective of regions, industries and enterprises. Third, we explore the reasons for the inverted U-shaped relationship between equity reform and high-quality development of state-owned enterprises. From the perspective of internal competition effect and capital speculation motivation, we further analyze the mediating mechanism of the impact of equity reform on the high-quality development of state-owned enterprises, and further verify that equity reform should adopt the principle of moderation.

## Theoretical Analysis and Hypotheses Development

Brandt et al. (2012) points out that the production and operation efficiency of state-owned enterprises is lower than that of non-state-owned enterprises. This suggests that non-government equity in an enterprise can improve the quality of state-owned enterprise development. First, the policy burden of state-owned enterprises is an important factor that affects production efficiency and development quality. The administrative management of state-owned assets can easily cause serious problems such as policy losses and soft budget constraints (Lin et al., 1998). Second, the existing corporate governance model has loopholes and moral hazard problems (Hart, 1995), which makes the principal-agent problem widespread. The agency cost will affect the management and operation efficiency and resource allocation efficiency of the enterprise, and then hinder the improvement of total factor productivity (Williamson, 1993; Kornai et al., 2003). Third, the lack of supervision and restraint in the business decision-making process under the situation of “one shareholder only” of state-owned equity makes the internal operation of enterprises lack vitality and responsibility, resulting in inefficient production and operation. Then the decision-makers of the enterprise have the motivation to lie and attribute the poor management to bear the policy burden, which seriously hinders the improvement of the development quality of the state-owned enterprises.

In general, the performance level of state-owned enterprises with non-state-owned capital shares is better than that of wholly state-owned enterprises (Astami et al., 2010; Boateng and Wei, 2017). Siqueira et al. (2009) found that the reform of the ownership structure of state-owned enterprises has a positive effect on the state-owned enterprises, mainly because the introduction of non-state-owned capital can alleviate a number of problems. For example, it reduces the policy burden and alleviates the problem of moral hazard, it reduces agency costs, promotes the improvement of total factor productivity (Boardman et al., 2016), and further improves the quality of enterprise development. The introduction of non-state-owned capital can increase the supervision on the state-owned shareholders, and therefore effectively alleviate the moral risk problems. In addition, the state-owned enterprises make full use of the complementary effect of capital, effectively exert the creativity and vitality of non-state-owned capital, which promotes the enhancement of the awareness of competition within the state-owned enterprises and further improves the production efficiency and development quality level of the enterprises. At the same time, although non-state-owned capital shares have positive promotion effect on the development quality of state-owned enterprises, the influence of the degree of non-state-owned capital shares on enterprise development will be moderate. Non-state-owned shareholders tend to have short-term profit-seeking intentions, while the state-owned equity can effectively restrain such intentions (Iannotta et al., 2013; Borisova et al., 2015; Acharya et al., 2016). Hence, when non-state-owned shareholders are in the leading and controlling position while state-owned shareholders are in the secondary balanced position, the industry may be controlled by non-state-owned shareholders for self-centered purposes. Complete privatization may lead to low production efficiency and capability (Shaoul, 1997; Molinos-Senante and Sala-Garridor, 2015). A moderately centralized and balanced equity structure can effectively improve enterprise governance level and operation efficiency (Bennedsen and Wolfenzon, 2000; Gomes et al., 2005). The excessive proportion of non-state-owned capital may lead to arbitrage, and negatively affect the quality of enterprise development. Therefore, the impact of equity reform on the development quality of state-owned enterprises may be inverted U-shaped. Therefore, we suggest that:

*Hypothesis 1*: There is an inverted U-shaped relationship between the equity reform and the high-quality development level of state-owned enterprises.

Heterogeneous factors, including the degree of regional marketization and financial development, not only affect the business model of enterprises, but also determine the degree of external factors’ influence on the changes of internal conditions of enterprises, and they then affect the quality level of enterprise development (Zhao and Wang, 2020). The reform and development of mixed ownership in China is stable, and the field of pilot reform has gradually spread to the whole country (Qian et al., 2008). For state-owned enterprises undergoing mixed ownership reform, the impact of the same proportion of equity structure on the development quality of state-owned enterprises may be different between economic developed regions and regions with high level of marketization (Wei et al., 2005). The impact of mixed ownership reform tends to be great on SOE development in regions with high level of marketization (Wei et al., 2003; Yuan et al., 2021), while the moderation effect of equity reform may exist in regions with low level of marketization. Thus, external market competition factors, such as the level of marketization and the regional market share of state-owned enterprises, profoundly affect the equity reform of state-owned enterprises. Due to the complementary effect between the market competition mechanism and the equity structure reform, the impact of equity reform on the development quality of state-owned enterprises may have regional heterogeneity. Therefore, for the state-owned enterprises in different regions, we put forward the following hypothesis:

*Hypothesis 2a*: The effect of equity reform on the development quality of state-owned enterprises has regional heterogeneity.

State-owned enterprises in different industry categories have different growth potential and social and economic responsibilities. Hence, the optimal proportion of the introduction of non-state-owned capital should be different. On the one hand, compared with commercial enterprises, China’s public welfare enterprises need to take certain social responsibilities and achieve the goal of stabilizing people’s livelihood. Therefore, it is necessary to have a considerable share of state-owned equity to ensure the efficient implementation of the government’s decisions on pricing and adjustment of supply and demand. On the other hand, in public welfare sector, the overall efficiency of state-owned enterprises is high (Shaoul, 1997). Although the introduction of non-state-owned capital can enhance enterprises’ internal vitality and innovation motivation (Boubakri et al., 2013) and thus optimize the corporate governance structure, the state-owned capital is still needed to solve the problems when facing financing constraints. The state-owned capital is easier to obtain government’s preferential policy support and trust from financial system. Therefore, for different types of state-owned enterprises, we put forward the following hypothesis:

*Hypothesis 2b*: The effect of equity reform on the development quality of state-owned enterprises has industry heterogeneity.

Modern enterprise theory holds that the corporate governance structure is a set of institutional arrangements used to dominate the relationship between relevant interest groups in the enterprise and thus realize the maximization of interests. Enterprises’ internal governance profoundly affects their execution of high-quality decisions after the equity reform (Jiang and Wang, 2017). Enterprises at higher development level usually have advanced governance structure and high efficiency of policy execution. Accordingly, for state-owned enterprises with different levels of development, we put forward the following hypothesis:

*Hypothesis 2c*: The effect of equity reform on the development quality of state-owned enterprises is affected by the development level of the enterprises themselves.

The introduction of non-state-owned capital has a two-sided impact on the quality of the development of enterprises in the process of mixed ownership reform. On the one hand, the introduction of non-state-owned capital widens the salary gap, which increases internal competitiveness, increases risky investment behavior (Megginson et al., 1994) and innovation motivation (Boubakri et al., 2013). On the other hand, the decision-making of state-owned enterprises is always consistent with the national interests (Ma and Peverelli, 2019), and the state-owned enterprises have a certain policy burden and redundancy employment (Huang and Sheng, 2012). The introduction of non-state-owned capital may lead to the downsizing of enterprises, which in turn reduces the role of state-owned enterprises in maintaining social stability and development (Bai et al., 2006). In addition, the non-state-owned capital is more profit-driven and speculative. Hence, it may blindly pursue the rate of return on capital and ignores the medium and long-term development strategic objectives of enterprises. This may slow down the process of high-quality development of enterprises. Therefore, we have following hypothesis:

*Hypothesis 3a*: Equity reform can promote the high-quality development of state-owned enterprises by enhancing the internal competitiveness of enterprises.

*Hypothesis 3b*: The equity reform can improve the speculation motivation of the enterprises, and lead the enterprises to blindly pursue the rate of return on capital, which hinders the high-quality development of the state-owned enterprises.

## Research Design

### Regression Model

We use the high-dimensional fixed effect model proposed by Correia et al. (2020) to set up a basic regression model, which aims to verify the effect of equity reform on the high-quality development level of state-owned enterprises and conduct multidimensional heterogeneity analysis, that is, verify Hypothesis 1 and Hypothesis 2.


(1) $$\begin{array}{l} hqd_{it}=\alpha_{0}+\partial_{1}mix_{it}+\partial_{2}w_{it}+\partial_{3}age_{it}+\partial_{4}ad_{it}\\ \;\;\;\;\;\;\;\;\;\;\;\;\;\;\;+\partial_{5}bsize_{it}+\partial_{6}pid_{it}+\partial_{7}pclevel_{it}\\ \;\;\;\;\;\;\;\;\;\;\;\;\;\;\;+\partial_{8}gender_{it}+\varepsilon_{it} \end{array}$$

(2) $$\begin{array}{l} hqd_{it}=\beta_{0}+\beta_{1}mix_{it}+\beta_{2}mix^{2}{}_{it}+\beta_{3}w_{it}+\\ \;\;\;\;\;\;\;\;\;\;\;\;\;\;\;\;\;\;\;\beta_{4}age_{it}+\beta_{5}ad_{it}+\beta_{6}bsize_{it}+\beta_{7}pid_{it}+\\ \;\;\;\;\;\;\;\;\;\;\;\;\;\;\;\;\;\;\;\beta_{8}pclevel_{it}+\beta_{9}gender_{it}+\varepsilon_{it} \end{array}$$

Among them, the *hqd* represents enterprises’ high-quality development level, and the *mix* represents the degree of equity reform, which is measured by the proportion of non-state-owned shares of the top five shareholders. The *w* represents the investment level of human capital of the enterprise, which is measured by the average wage of the employees of the enterprise. The *age* represents the listing period of an enterprise and is measured logarithmically by the difference between the current time and the listing time. The *ad* represents advertising expenses, measured as the proportion of sales expenses to operating income. The *bsize* represents the size of the board, measured by the number of board members. The *pid* represents the size of independent directors, measured by the proportion of independent directors. The *pclevel* represents the political connection degree of decision-making level measured by commonly used set sequencing variables. The *gender* represents the gender heterogeneity of top management team, measured by Herfindal–Hirschman coefficient.^2^ The *ε* is a random interference term.

We build regression models of Equations 3, 4 according to the mediate effect step-by-step analysis method proposed by Baron and Kenny (1986), which explores the transmission mechanism of the impact of equity reform on the high-quality development of state-owned enterprises with the combination of Equation 1, that is, verify Hypothesis 3a and Hypothesis 3b.


(3) $$gap_{it}\;|tobin_{it}=\gamma_{0}+\gamma_{1}mix_{it}+\overset{8}{\underset{j=2}{\sum }}\gamma_{j}control_{jit}+\varepsilon_{it}$$

(4) $$hqd_{it}=\phi_{0}+\phi_{1}mix_{it}+\phi_{2}gap_{it}\;|tobin_{it}+\overset{9}{\underset{j=3}{\sum }}\phi_{j}control_{jit}+\varepsilon_{it}$$

### Variables Description

#### The Indicators of the Level of High-Quality Development in State-Owned Enterprises

The 19th National Congress of the Communist Party of China (CPC) reported that “we will further reform state-owned enterprises, develop mixed-ownership economic entities, and turn Chinese enterprises into world-class.” The 16th Plenary Session of the 19th Central Committee also clearly pointed out that “we will support state-owned capital and state-owned enterprises to become stronger, do better and growth bigger, and establish a modern enterprise system with Chinese characteristics, and strengthen the competitiveness, innovation, control, influence and risk resistance of the state-owned economy.” Obviously, the purpose of deepening the reform of state-owned enterprises is to make the state-owned capital of state-owned enterprises becoming stronger, doing better, and growing bigger, so as to effectively improve the high-quality development level of state-owned enterprises and cultivate world-class enterprises with global competitiveness. Therefore, the criteria of “becoming stronger, doing better, and growing bigger” is the result of the CPC definition of high-quality development. Accordingly, the paper constructs the high-quality development index of state-owned enterprises by a principal component analysis from the perspectives of innovation level, total factor productivity, enterprise growth, production and operation efficiency and enterprise scale. The specific indicators are shown in Table 1, while the weight information of each principal component after principal component analysis is shown in Table 2.

**Table 1:** Index of high-quality development level of state-owned enterprises in the new era.

First-level indicators	Second-level indicators	Third-level indicators	Notes
The high-quality development level of state-owned enterprises in the new era.	Becoming stronger	Innovation level	Number of patents authorized
			Number of green patent licenses
		Total factor productivity	LP semi-parametric calculation (Levinsohn and Petrin, 2003)
	Doing better	Growth	Total assets growth rate
			Operating income growth rate
		Production and operation efficiency	Return on net assets
			Total assets profit margin
			Labor efficiency (operating income/employee count)
		Corporate soft power	Net intangible assets
		Corporate social responsibility	The data of corporate social responsibility index are from Hexun.com^1^
	Growing bigger	Scale	Operating income
			Net assets per share
			Net profit

1 *http://stockdata.stock.hexun.com/zrbg/Plate.aspx.*

**Table 2:** The weight of each principal component after principal component analysis.

Year	The value of KMO^*^	Principal component contribution rate (%)							Cumulative contribution rate (%)
		1	2	3	4	5	6	7	
2013	0.637	26.26	13.32	12.57	9.67	8.65	6.64	6.33	83.44
2014	0.607	24.32	16.49	10.14	8.58	8.32	7.41	5.72	80.98
2015	0.661	24.79	14.63	12.36	9.35	8.49	6.02	5.72	81.37
2016	0.657	24.22	15.09	11.93	10.12	7.93	7.09	6.17	82.55
2017	0.685	25.03	14.61	10.26	8.49	8.36	7.49	6.67	80.91
2018	0.668	25.86	16.08	10.62	8.67	7.93	6.98	6.02	82.16

* *The KMO value of each year is greater than 0.6, indicating that the basic index is suitable for principal component analysis*.

#### The Measurement of Equity Reform

The paper uses *mix* to express the degree of equity reform and is measured by the proportion of non-state-owned shares among the top five shareholders of the enterprise, which can reflect the changes in equity situation of state-owned enterprises in the process of non-state-owned capital introduction. The *mixx* is as an alternative variable of equity reform in the robustness checks. According to the nature of the top five shareholders of the enterprise, the paper calculates the proportion of non-state-owned equity (*nGy*) and state-owned equity (*Gy*) to indicate the degree of equity reform in state-owned enterprises (when *nGy* > *Gy*, *mixx* = *Gy*/*nGy*, when *Gy* > *nGy*, *mixx* = *nGy*/*Gy*). The *mixxx* is also as an alternative variable of equity reform in the robustness checks, which is measured by dividing the proportion of non-state-owned shares and state-owned shares by 100.

#### Description of Mediating Variables and Control Variables

First, according to Tournament Theory, employees’ salary can reflect their job position levels. Staff motivation in an enterprise is mainly affected by the salary gap between the level at which they are located and the higher level (Lazear and Rosen, 1981; Rosen, 1986). In other words, the salary gap can enhance the vitality and competitiveness of employees within the enterprise (Xu et al., 2017). Second, TobinQ measures the market performance of enterprises. Although the intrinsic relationship between TobinQ and corporate value is supported by foreign theoretical models and empirical evidence (Demsetz and Lehn, 1985; Morck et al., 1988), it is not applicable in China where there are differences in capital markets, and is mainly influenced by speculation, which is closely related to TobinQ (Huang et al., 2009). According to the Economic Human Assumption (Zhu and Liu, 2021), when TobinQ value is high, the enterprise will choose to convert financial capital into industrial capital, and when TobinQ value is low, the enterprise will choose to convert industrial capital into financial capital. Non-state-owned capital tends to be short-sighted and seeks maximum profit. Corresponding measures should be used according to the change of TobinQ value to affect the decision-making of enterprises. In the paper, the mediating variables are internal competitive atmosphere (*gap*) and profit-seeking speculative motivation (*tobin*). The *gap* was measured by the logarithm of the difference between the average salary of the top three executives and the average salary of the employees. The *tobin* was measured by the enterprise TobinQ. The empirical analysis also involves control variables, including the level of human capital investment (*w*), age of being listed (*age*), advertising expenses (*ad*), the size of the board (*bsize*), the size of independent directors (*pid*), the political connection degree of decision-making level (*pclevel*) and the gender heterogeneity of top management team (*gender*).

### Data Sources

This paper mainly explores the issues related to the impact of equity reform on the high-quality development of state-owned enterprises in the new era. According to the principle of data availability, the paper selects relevant data of A-share listed state-owned enterprises from 2013 to 2018 in CSMAR database and Wind database. First, according to the nature of corporate equity, the enterprises are divided into four categories: state-owned enterprises, private enterprises, foreign-funded enterprises and other enterprises. And the state-owned enterprises are selected. Second, due to the significant differences between financial service enterprises and other types of enterprises in terms of capital structure, operating methods and finance (Tian and Estrin, 2008). This paper further excludes financial service related enterprises. Third, this paper excludes enterprises that receive special treatment and the observation samples with serious data deficiency. Finally, The main variables are subjected to 1% tail reduction processing to eliminate the impact of extreme values. Hence, we selected 904 state-owned enterprises as our research targets which formed 5,424 observed units. The descriptive statistics of relevant variables are shown in Table 3.

**Table 3:** Descriptive statistics of the related variables.

VarName	Obs	Mean	Median	SD	Min	Max
*hqd*	5,398	−0.010	−0.088	0.524	−1.290	2.363
*tfp*	5,206	8.486	8.391	1.074	6.062	11.044
*rn*	2,558	12.587	10.185	11.767	0.120	62.450
*mix*	5,424	0.244	0.109	0.300	0.000	1.000
*mixx*	5,424	0.187	0.081	0.239	0.000	0.957
*mixxx*	5,025	0.010	0.001	0.042	0.000	0.327
*tobin*	5,271	1.916	1.468	1.296	0.850	8.587
*gap*	5,406	13.228	13.210	0.673	11.520	15.135
*w*	5,399	9.437	9.552	1.141	5.546	11.871
*age*	5,424	2.634	2.833	0.497	1.099	3.258
*ad*	5,424	5.163	3.153	6.315	0.000	33.889
*bsize*	5,423	9.116	9.000	1.828	5.000	15.000
*pid*	5,422	0.373	0.357	0.056	0.333	0.600
*pclevel*	5,424	0.933	0.000	1.467	0.000	4.000
*gender*	5,423	0.177	0.198	0.178	0.000	0.500

## Regression Analysis

### Baseline Regression Analysis

We used a gradual regression for Equations 1, 2 to test the specific effect of the equity reform on the high quality of the development of state-owned enterprises. The results are shown in Table 4. Models 1 and 2 are regression results of enterprise fixed effect model and two-way fixed effect model, respectively, without adding control variables. Model 3 is the result after adding control variables, while model 4 is the result after further adding the quadratic term of equity reform. It can be seen from Table 3 that before and after adding control variables, the effect of equity reform on the high quality of the development of state-owned enterprises is significantly positive and has passed the 1% confidence level test. After the control variables were added, the primary term and the quadratic term of equity reform are positive and negative, respectively. Both of them passed the confidence level test below 5%, which reflects that the impact of equity reform on the development quality is changing as an inverted U-shape. There may be two main reasons for these results: first, the effects of the introduction of non-state-owned capital can be different in different regions, industries and development phases. Second, while the introduction of non-state-owned capital reduces the negative effect of policy burden on enterprises and alleviates the conflict of interests, other problems emerge such as speculation and arbitrage due to the imperfect institutional mechanism of enterprise capital regulation. This may lead to a decreasing positive marginal effect on high-quality development with the introduction of non-state-owned capital.

**Table 4:** Baseline regression result.

	(1)	(2)	(3)	(4)
	*hqd*	*hqd*	*hqd*	*hqd*
*mix*	0.188^***^	0.189^***^	0.190^***^	0.436^***^
	(0.050)	(0.050)	(0.050)	(0.117)
*mix* ^2^				−0.285^**^
				(0.122)
*w*			0.075^***^	0.076^***^
			(0.009)	(0.009)
*age*			−0.049	−0.058
			(0.059)	(0.059)
*ad*			−0.019^***^	−0.019^***^
			(0.002)	(0.002)
*bsize*			0.025^***^	0.024^***^
			(0.007)	(0.007)
*pid*			0.106	0.110
			(0.170)	(0.170)
*pclevel*			0.020^***^	0.020^***^
			(0.005)	(0.005)
*gender*			0.041	0.035
			(0.054)	(0.054)
*constant*	−0.055^***^	−0.056^***^	−0.834^***^	−0.822^***^
	(0.013)	(0.013)	(0.207)	(0.207)
Enterprise fixed	YES	YES	YES	YES
Time fixed		YES	YES	YES
*N*	5,398	5,398	5,371	5,371
*R* ^2^	0.659	0.660	0.671	0.672

*The values in brackets are SDs*.

*** *Indicates that the estimated coefficients are significant at the confidence levels of 1%*.

** *Indicates that the estimated coefficients are significant at the confidence levels of 5%*.

* *Indicates that the estimated coefficients are significant at the confidence levels of 10%*.

To test the robustness, we replaced the analytical model, the high-quality development indicators of enterprises and the measurement method of equity reform. The results are shown in Table 5. Model 1 is the regression result of the Tobit model. The inverted U-shaped relationship between equity reform and high-quality enterprise development exists significantly at 1% confidence level. On the one hand, the high-quality of the development of state-owned enterprises requires innovation as the core driving force. And the status and importance of enterprise innovation development largely represent the competitiveness and high-quality development level of enterprises (Akbari et al., 2021; Georgieva and Georgieva, 2022). Therefore, Model 2 uses the importance of enterprise innovation (it is measured by the proportion of enterprise research and development staff) as an alternative variable for high-quality development for robustness test. The regression results show that the inverted U-shaped effect of equity reform is still significant at the confidence level of 5%. On the other hand, the total factor productivity of an enterprise is usually taken as an important indicator to measure the development level of an enterprise, and the key to high-quality development in an enterprise is also an improvement in the total factor productivity. The model 3 uses total factor productivity as an alternative variable for the robustness test. The regression results show that the inverted U-shaped effect of equity reform exists significantly at the confidence level of 1%. Based on the characteristics of equity reform, the paper uses the mixed degree index and the ratio of non-state-owned equity to state-owned equity instead of the proportion of non-state-owned shares as the core explanatory variable to test the robustness. The regression results of model 4 and model 5 support the conclusion of the inverted U-shaped effect.

**Table 5:** The robustness checks.

	(1)	(2)	(3)	(4)	(5)
	*hqd*	*inno*	*tfp*	*hqd*	*hqd*
*mix*	0.491^***^	6.318^**^	0.322^***^		
	(0.100)	(2.521)	(0.106)		
*mix* ^2^	−0.458^***^	−7.373^***^	−0.296^***^		
	(0.103)	(2.481)	(0.110)		
*mixx*				0.318^***^	
				(0.103)	
*mixx* ^2^				−0.225^*^	
				(0.117)	
*mixxx*					0.316^**^
					(0.154)
*mixxx* ^2^					−0.086^*^
					(0.045)
*constant*	−1.022^***^	22.097^***^	6.884^***^	−0.758^***^	−1.277^***^
	(0.121)	(5.903)	(0.187)	(0.206)	(0.233)
*control*	YES	YES	YES	YES	YES
Enterprise fixed	YES	YES	YES	YES	YES
Time fixed	YES	YES	YES	YES	YES
$\sigma_{u}$	0.385^***^				
	(0.010)				
$\sigma_{e}$	0.331^***^				
	(0.004)				
Wald test	244.83^***^				
*R* ^2^		0.919	0.939	0.671	0.689
*N*	5,372	2,503	5,179	5,371	4,954

*The values in brackets are SDs*.

*** *Indicates that the estimated coefficients are significant at the confidence levels of 1%*.

** *Indicates that the estimated coefficients are significant at the confidence levels of 5%*.

* *Indicates that the estimated coefficients are significant at the confidence levels of 10%*.

The paper eliminates the endogeneity problem through the tool variable method, and selects the tool variable by using the “industry average” method and the core explanatory variable lag term. Specifically, we use the IV-two-stage least squares regression (IV-2SLS) method, optimal generalized methods of moments (GMM) and the iterative GMM to alleviate the potential endogeneity issue. We chose the proportion of equity reform of state-owned enterprises in the industry (*mix_ind*) and the degree of lagged one-period equity reform (*L.mix*) as the instrumental variables, and the results of IV-2SLS, GMM and IGMM are shown for model 1–3, respectively, in Table 6. The value of *p* of over-identification test is 0.613, which accepts the assumption of “all tool variables are exogenous,” that is, the proportion of equity reform of state-owned enterprises in the industry (*mix_ind*) and the degree of lagged one-period equity reform (*L.mix*) are exogenous. As Shea’s partial *R*^2^ is 0.355, value of *p* is 0.000 for the *F*-test and the minimum Eigenvalue statistic is1225.72 (much more than 10), the hypothesis of “weak instrumental variables” was rejected. The results from model 1 to model 3 show that the regression coefficients of the primary term and quadratic term of equity reform are still significant at the 1% confidence level, and the results of the optimal GMM and iterative GMM are basically consistent with those of IV-2SLS. The inverted U-shaped relationship between equity reform and the high quality of the development of state-owned enterprises is further verified. Additionally, the paper takes the lagging high-quality development level as the explanatory variable to eliminate the endogenous influence of the inertia effect of high-quality development on regression analysis. The regression results are shown in model 4, and the inverted U-shaped relationship exists significantly within the confidence level of less than 10%. In conclusion, the inverted U-shaped relationship is robust. In other words, the equity reform has an ever-changing role in promoting high-quality development in state-owned enterprises. Hypothesis 1 is verified.

**Table 6:** Endogeneity test.

	(1)	(2)	(3)	(4)
	*iv-2sls*	*gmm*	*igmm*	*L.hqd*
*mix*	0.482^***^	0.478^***^	0.478^***^	0.466^***^
	(0.178)	(0.178)	(0.178)	(0.133)
*mix* ^2^	−0.539^***^	−0.537^***^	−0.537^***^	−0.223^*^
	(0.175)	(0.175)	(0.175)	(0.127)
*L.hqd*				−0.040^**^
				(0.017)
*constant*	−1.378^***^	−1.379^***^	−1.379^***^	−0.453^*^
	(0.112)	(0.112)	(0.112)	(0.266)
*control*	YES	YES	YES	YES
*Test of overidentifying restrictions*	0.256	0.256	0.256	
	[0.613]	[0.613]	[0.613]	
*Shea’s partial R* ^2^	0.355			
*Robust F*	191.638			
	[0.000]			
*Minimum eigenvalue statistic*	1225.72			
*R* ^2^	0.078	0.078	0.078	0.708
*N*	4,479	4,479	4,479	4,458

*The values in brackets “( )” are SDs. The values in brackets “[ ]” are the value of *p* of the corresponding test statistics. The values in brackets are SDs*.

*** *Indicates that the estimated coefficients are significant at the confidence levels of 1%*.

** *Indicates that the estimated coefficients are significant at the confidence levels of 5%*.

* *Indicates that the estimated coefficients are significant at the confidence levels of 10%*.

### Heterogeneity Analysis

The reform of mixed ownership of state-owned enterprises is dynamically different due to the differences in market environments and level of self-development caused by different geographical regions, industries and enterprise characteristics. Due to the complementary effects between market-based competitive mechanisms and equity structure reform, the impact of equity reform on the development quality of state-owned enterprise may be heterogeneous in multiple dimensions. To test whether there is heterogeneity in the impact of equity reform on the high quality of the development of state-owned enterprises, the paper conducts grouping empirical test from the perspective of time, regional, industry and enterprise category heterogeneity.

Since the 18th National Congress of the Communist Party of China in 2013, relevant policies have been issued one after another to support high-quality sustainable development of the national economy and the development of the mixed ownership economy. The state-owned capital supervision department and the state-owned enterprises are steadily pushing forward the reform of mixed ownership in the new era and establishing a modern state-owned enterprise system. The “Guiding Opinions on Deepening the Reform of State-owned Enterprises” issued in 2015 has further pushed the reform to a new level. Therefore, the paper takes 2015 as the cut-off point to empirically test the impact of equity reform on the high-quality development of state-owned enterprises in the two time periods, 2013–2015 and 2016–2018. The regression results are shown in Table 7. According to models 1 and 2, the regression coefficient of equity reform is not significant before adding the quadratic term. After adding the quadratic term, the effect of equity reform on the high-quality development of state-owned enterprises is an inverted U-shaped and has passed the confidence level test below 5%. This indicates that during 2013–2015, the effect of the reform on the quality was not high, and the reform dividend was not released effectively. According to models 3 and 4, the regression coefficient of equity reform is significantly positive within the 1% confidence level before adding the quadratic term, and the quadratic term coefficient is not significant after adding the quadratic term. It shows that the quality of equity reform improved, the promotion effect showed a monotonic increasing trend from 2016 to 2018 and the benefits of the mixed reform increased steadily after 2015 when more a specific reform policy was issued.

**Table 7:** The analysis of the temporal heterogeneity.

	(1)	(2)	(3)	(4)
	*hqd*	*hqd*	*hqd*	*hqd*
*mix*	0.081	0.464^**^	0.255^***^	0.491^**^
	(0.101)	(0.194)	(0.095)	(0.241)
*mix* ^2^		−0.540^**^		−0.251
		(0.233)		(0.234)
*constant*	−0.806^**^	−0.817^**^	−2.777^***^	−2.683^***^
	(0.375)	(0.374)	(0.689)	(0.695)
*control*	YES	YES	YES	YES
Enterprise fixed	YES	YES	YES	YES
Time fixed	YES	YES	YES	YES
*N*	2,674	2,674	2,692	2,692
*R* ^2^	0.772	0.773	0.756	0.757

*The values in brackets are SDs*.

*** *Indicates that the estimated coefficients are significant at the confidence levels of 1%*.

** *Indicates that the estimated coefficients are significant at the confidence levels of 5%*.

* *Indicates that the estimated coefficients are significant at the confidence levels of 10%*.

As shown in Table 8, the equity reform has a significant effect on promoting the high-quality development of state-owned enterprises in each region and has passed the confidence level test of less than 10%. From the perspective of moderation, it was found that only the northeast region passes the significance test. This may be due to the difference in the proportion of local state-owned enterprises in different regions and the different degree of influence on the quality of the development as a result of the equity reform. The quadratic regression coefficients for equity reform in other regions are not significant. This indicates that the benefits of equity reform are still relatively evident in all regions during the sample period, and it is important to promote equity reform. In addition, the regression coefficient of the equity reform in the eastern and central regions are smaller, while the regression coefficient of the equity reform in the western and northeast regions on the quality of enterprise development are larger. This indicates that there are regional differences in the effects of the equity reform. The main reason may be that the effect of equity reform is different under the added effect of external market competition mechanisms and internal equity structure reforms. The state-owned enterprise equity reform in the eastern and central regions with a high degree of market-oriented competition has a relatively low impact on the quality of enterprise development. However, the degree of marketization in the western and north-eastern regions is relatively low, the market share of state-owned enterprises is relatively high, thus the equity reform of state-owned enterprises affects both the market operation and the development of enterprises. The optimized market environment further promotes the quality of development, making the effect of equity reform obvious. In other words, the effect of equity reform has regional heterogeneity. Hence, the Hypothesis 2a is verified.

**Table 8:** The analysis of regional heterogeneity.

	The eastern	The central	The western	The north-eastern	The eastern	The central	The western	The north-eastern
	*hqd*	*hqd*	*hqd*	*hqd*	*hqd*	*hqd*	*hqd*	*hqd*
*mix*	0.199^***^	0.198^*^	0.278^***^	0.391^***^	0.394^***^	0.120	0.335	1.120^***^
	(0.066)	(0.105)	(0.104)	(0.144)	(0.152)	(0.242)	(0.253)	(0.404)
*mix* ^2^					−0.215	0.097	−0.074	−1.185^***^
					(0.151)	(0.268)	(0.299)	(0.414)
*constant*	−1.260^***^	0.389	−0.193	0.362	−1.240^***^	0.395	−0.196	0.183
	(0.252)	(0.474)	(0.524)	(0.889)	(0.252)	(0.474)	(0.525)	(0.878)
*control*	YES	YES	YES	YES	YES	YES	YES	YES
Enterprise fixed	YES	YES	YES	YES	YES	YES	YES	YES
Time fixed	YES	YES	YES	YES	YES	YES	YES	YES
*N*	3,071	981	1,012	307	3,071	981	1,012	307
*R* ^2^	0.696	0.618	0.534	0.547	0.696	0.618	0.534	0.562

*The values in brackets are SDs*.

*** *Indicates that the estimated coefficients are significant at the confidence levels of 1%*.

** *Indicates that the estimated coefficients are significant at the confidence levels of 5%*.

* *Indicates that the estimated coefficients are significant at the confidence levels of 10%*.

There are differences in the industry development environment. Hence, state-owned enterprises may have different self-positioning for different industries, which may lead to different effects due to equity reform. The paper classifies the state-owned enterprises into commercial and public welfare categories according to the relevant policy guidelines such as “Guidance on Deepening the Reform of State-owned Enterprises” in 2015 and “Industry Classification Guide for Listed Companies” in 2012 (See Appendix one). Hence, we explored the heterogeneous impact of equity reform on the development quality of state-owned enterprises. The regression results are shown in Table 9. Models 1, 2 and 3, 4 are regression results of public welfare state-owned enterprises and commercial state-owned enterprises, respectively. It can be seen that the equity reform has a positive promoting effect on the high-quality development of public welfare state-owned enterprises and commercial state-owned enterprises. Such effect is significant under the confidence level of 10%, but the regression coefficient of commercial state-owned enterprises is higher and the effect is more significant than public welfare enterprises. The regression coefficients of the primary term and the quadratic term of the equity reform in public welfare enterprises are one positive and one negative, respectively. The relationship between the equity reform of state-owned enterprises and the high-quality development of enterprises is changing significantly as an inverted U-shape. The quadratic coefficient of equity reform in commercial enterprises is negative, but not significant, which indicates that the impact of equity reform on the quality of the development of commercial state-owned enterprises mainly shows a positive effect. Therefore, the effect of equity reform on the development of state-owned enterprises has industry heterogeneity. For the public welfare state-owned enterprises, the principle of proportionality should be emphasized in the process of introducing non-state-owned capital, so as to effectively prevent the excessive reform. For the commercial state-owned enterprises, actively promoting the equity reform process is suggested for improving enterprise development quality. Hence, Hypothesis 2b is verified.

**Table 9:** The analysis of the industry heterogeneity.

	(1)	(2)	(3)	(4)
	*hqd*	*hqd*	*hqd*	*hqd*
*mix*	0.136^*^	0.455^**^	0.214^***^	0.428^***^
	(0.080)	(0.187)	(0.064)	(0.149)
*mix* ^2^		−0.382^*^		−0.244
		(0.203)		(0.154)
*constant*	−1.444^***^	−1.411^***^	−0.518^*^	−0.516^*^
	(0.335)	(0.335)	(0.265)	(0.265)
*control*	YES	YES	YES	YES
Enterprise fixed	YES	YES	YES	YES
Time fixed	YES	YES	YES	YES
*R* ^2^	0.679	0.679	0.669	0.670
*N*	2050	2050	3,321	3,321

*The values in brackets are SDs*.

*** *Indicates that the estimated coefficients are significant at the confidence levels of 1%*.

** *Indicates that the estimated coefficients are significant at the confidence levels of 5%*.

* *Indicates that the estimated coefficients are significant at the confidence levels of 10%*.

In addition, the effect of equity reform may be different at different development phases. The paper uses a quantile regression model to select 10%, 25%, 75%, and 90% for regression analysis to verify the impact of equity reform on high-quality development in enterprises at different development phases. As shown in Table 10, In general, the regression coefficient of the equity reform from 10% to 90% is constantly increasing, and are all significantly positive at the confidence level of 1%. This indicates that the benefits of equity reform on the quality of enterprise development becomes obvious when enterprises develop into more advance phases. The enterprises in advanced phases of development have better operation mechanisms, and the benefits of equity reform can be better released. It is worth noting that when the development level of state-owned enterprises is below 10%, the regression coefficient of equity reform on the development quality of state-owned enterprises is relatively large (0.151). The state-owned enterprises with low level of development are suggested to promote the equity reform. Hence, the effect of equity reform on the development quality of state-owned enterprises is obvious. In addition, such effect has development-level heterogeneity. Hypothesis 2c is verified.

**Table 10:** The analysis of development-level heterogeneity.

	(10%)	(25%)	(50%)	(75%)	(90%)
	*hqd*	*hqd*	*hqd*	*hqd*	*hqd*
*mix*	0.151^***^	0.117^***^	0.133^***^	0.212^***^	0.234^***^
	(0.032)	(0.020)	(0.019)	(0.038)	(0.065)
*control*	YES	YES	YES	YES	YES
Enterprise fixed	YES	YES	YES	YES	YES
Time fixed	YES	YES	YES	YES	YES
*N*	5,373	5,373	5,373	5,373	5,373

*The values in brackets are SDs*.

*** *Indicates that the estimated coefficients are significant at the confidence levels of 1%*.

** *Indicates that the estimated coefficients are significant at the confidence levels of 5%*.

* *Indicates that the estimated coefficients are significant at the confidence levels of 10%*.

### The Analysis of Transmission Mechanism

This section further tests the transmission mechanism of the effect of equity reform and verifies whether or not the introduction of non-state-owned capital will affect the quality of development in state-owned enterprises by creating an atmosphere of internal competition, profit-seeking and speculation. We used Baron and Kenny (1986) mediating effect step-by-step analysis method to regress Equations 3, 4 in order to explore the transmission mechanism of the impact of equity reform on the development of state-owned enterprises based on the benchmark regression results.

As shown in Table 11, the regression coefficient of equity reform on internal competitive incentives is significantly positive at the confidence level of 5%. In addition, the effects of equity reform and internal competitiveness on high-quality development of enterprises are significantly positive at the confidence level of 1%. This indicates that the equity reform can activate internal vitality through encouraging internal competitiveness and improving the quality of development. The positive mediating effect is 0.015 (0.129 × 0.113). From the regression results of the intermediary effect of profit-seeking and speculation, we can see that the regression coefficient of equity reform on profit-seeking and speculation is significantly positive under the confidence level of 1%, while the regression coefficient of profit-seeking and speculation on high-quality development of enterprises is significantly negative under the confidence level of 1%, indicating that the introduction of non-state-owned capital can increase internal profit-seeking and speculation while ignoring strategic development planning and slowing down the process of high-quality development. The negative intermediary effect is −0.010 (0.396 × −0.026). Moreover, the Sobel test showed that the mediating effect of internal competition incentives and profit-seeking speculation both pass the significance test of 5% confidence level. Therefore, there is a two-fold impact of the introduction of non-state-owned capital on the development of the enterprise in the process of equity reform. While making good use of the comparative advantages of non-state-owned capital, its negative impact of profit-seeking and speculation should not be neglected. Hence, the Hypothesis 3a and Hypothesis 3b are verified.

**Table 11:** The regression result of mediate effect based on internal competition incentive and capital speculation motivation.

	(1)	(2)	(3)	(4)
	*gap*	*hqd*	*tobin*	*hqd*
*mix*	0.129^**^	0.205^***^	0.396^***^	0.203^***^
	(0.057)	(0.046)	(0.107)	(0.050)
*gap*		0.113^***^		
		(0.012)		
*tobin*				−0.026^***^
				(0.007)
*constant*	13.079^***^	−1.912^***^	1.745^***^	−0.829^***^
	(0.202)	(0.227)	(0.439)	(0.203)
*control*	YES	YES	YES	YES
Enterprise fixed	YES	YES	YES	YES
Time fixed	YES	YES	YES	YES
*N*	5,385	5,360	5,243	5,220
*R* ^2^	0.828	0.623	0.763	0.689
Sobel test	0.015^**^	(*z* = 2.250, *p* = 0.024)	−0.010^**^	(*z* = −2.476, *p* = 0.011)

*The values in brackets are SDs*.

*** *Indicates that the estimated coefficients are significant at the confidence levels of 1%*.

** *Indicates that the estimated coefficients are significant at the confidence levels of 5%*.

* *Indicates that the estimated coefficients are significant at the confidence levels of 10%*.

## Conclusions and Policy Implications

### Conclusion

Equity reform can improve the quality of the development of state-owned enterprises. The overall effect is an inverted U-shaped with multi-dimensional heterogeneity. From the perspective of time heterogeneity, the implementation of the classified reform policy for state-owned enterprises in 2015 effectively corrected the excessive equity reform and enhanced the effect of the equity reform. From the perspective of regional heterogeneity, the equity reform in the western and north-eastern regions has a more obvious effect on high-quality development than that of eastern and central region. The state-owned enterprises in the western and north-eastern regions have a large market share, which has a high degree of influence on marketization. The acceleration of marketization further promotes the improvement of enterprise development quality and forms a virtuous circle. From the perspective of industry heterogeneity, the equity reform of public-interest state-owned enterprises has an inverted U-shaped relationship with the development quality. In addition, the positive effect of equity reform can be found in commercial state-owned enterprises. Such effect is monotonic and prominent due to the principle of competitiveness. From the perspective of heterogeneity of enterprise development quality, a stronger positive effect of equity reform can be found in enterprises at advanced development phase with a mature enterprise system than those in a less advanced development phase.

The introduction of non-state-owned capital in the process of equity reform has a dual impact on high-quality development. The addition of non-state-owned capital promotes the internal competitiveness, which further improves the quality of development. This indicates that the internal competitive atmosphere serves as a significant positive intermediary effect. Additionally, due to the profit-seeking characteristics of non-state-owned capital, the increase in the proportion of non-state-owned capital encourages enterprises to speculate and slows down high-quality development. In other words, profit-seeking and speculation with private capital in the enterprise show a significant negative intermediary effect.

### Policy Implications

On the one hand, we should clearly understand the appropriateness of equity reform, and implement policies based on local conditions, industry and enterprises, so as to effectively promote the high quality of the development of state-owned enterprises. Compared with complete privatization, partial privatization of state-owned enterprises is more conducive to high-quality development. According to the characteristics of state-owned enterprises in different regions, different industries and different development phases, exclusive equity reform should be carried out to promote steady enterprise development. First, attention should be given to regional differences in terms of market environment. Regions that have a large number of state-own enterprises are affected more by equity reform. Regionally different market environments can further affect state-owned enterprise development. Guiding the positive impact of equity reform to stimulate high-quality development becomes important. Second, according to the characteristics of different industries, the relevant departments should effectively formulate the equity reform plan. Effective equity reform plans should be formulated according to the nature and characteristics of local industries. While introducing non-state-owned capital to improve vitality and competitiveness, the public welfare state-owned enterprises should pay attention to the changing impact of the reform to protect their rights and control for high-quality development. Commercial state-owned enterprises should pay attention to the principle of competitiveness in the process of equity reform, and actively carry out equity reform to improve the quality of development. Third, the enterprises at advanced development phases should effectively take advantage of mixed ownership capital through the equity reform based on their relatively mature internal operation mechanisms and consummate system, so as to realize the constancy of the strong for the enterprises with higher level of development.

On the other hand, we should improve the capital regulatory system to supervise the endowment structure and comparative advantages of non-state-owned capital to achieve high-quality development of state-owned enterprises. First, the characteristics of private capital, such as a propensity for taking higher risks and more competitive vitality should be converted to a positive intermediary effect of internal competitiveness, which increases internal vitality and development enthusiasm for operation efficiency and quality. Second, a capital supervision system should focus on the profit-seeking characteristics of non-state-owned capital and prevent negative intermediary effect of speculative behavior. Third, the difference between strategic and profit-seeking capital should be addressed. For building a cooperative working environment and win-win benefit, non-state-owned capital should be selectively introduced according to state-owned strategic plan of the state-owned enterprise.

## Data Availability Statement

The original contributions presented in the study are included in the article/supplementary material; further inquiries can be directed to the corresponding authors.

## Author Contributions

HL reviewed relevant literature, supervised the work, and acquired fund for the research. DW provided most of the writing—review and editing, and made the original draft preparation. LZ developed the conceptualization and research methodology. JZ analyzed the data and made calculation. All authors contributed to the article and approved the submitted version.

## Funding

This research was supported by the Soft Science Research Program under the Science and Technology Department of Jilin Province (grant number: 20190601084FG), the Science Research Program under the Education Department of Jilin Province (grant number: JJKH20211239SK), the Xi’an Eurasia University Technological Service Special Program (grant number: OYJSFW-2021001), and the National Social Science Funding “Research on Transformation of State-Owned Assets Supervision from ‘Managing Assets’ to ‘Managing Capital’” (grant number: 17BJY164).

## Conflict of Interest

The authors declare that the research was conducted in the absence of any commercial or financial relationships that could be construed as a potential conflict of interest.

## Publisher’s Note

All claims expressed in this article are solely those of the authors and do not necessarily represent those of their affiliated organizations, or those of the publisher, the editors and the reviewers. Any product that may be evaluated in this article, or claim that may be made by its manufacturer, is not guaranteed or endorsed by the publisher.

## Figures and Tables

**Table:** 

Industry sort	Industry code	Industry name
Public welfare state-owned enterprises	B06–B09	B06: Coal mining and washing industry. B07: Oil and gas mining industry. B08: Ferrous metal mining and dressing industry. B09: Non-ferrous metal mining and dressing industry.
	C25, C31–C32, C34, C37	C25: Petroleum processing, coking, and nuclear fuel processing industry; C31: Black metal smelting and calendering industry; C32: Non-ferrous metal smelting and caldering industry: C34: General equipment manufacturing industry; C37: Railway, shipping, aerospace and other transportation equipment manufacturing industry
	D44–D46	D44: Power and heat production and supply industry; D45: Gas production and supply industry; D46: Water production and supply industry
	E48	E48: Civil Engineering construction industry
	G53–G55, G58–G59	G53: Railway transportation industry; G54: Road transport industry; G55: Water transportation industry; G58: loading, unloading, handling and transportation agency industry; G59: Storage industry
	I63	I63: Telecommunications, radio and television, and satellite transmission services
	N77–N78	N77: Ecological protection and environmental governance industry; N78: Public facilities management industry
	P82	P82: Education
	R85–R86	R85: Journalism and publishing industry; R86: Radio, television, film and film recording production industry
	S90	S90: Comprehensive
Commercial state-owned enterprises	A01–A05	A01: Agriculture; A02: Forestry; A03: Animal husbandry; A04: Fisheries; A05: Agriculture, forestry, animal husbandry and fishery services	
	B11	B11: Mining auxiliary activities	
	C13–C15, C17–C18, C20, C22–C24, C26–C30, C33, C35–C36, C38–C40, C42	C13: Agricultural and sideline food processing industry; C14: Food manufacturing industry; C15: Wine, beverage and refined tea manufacturing industry; C17: Textile industry; C18: Textile and clothing, clothing industry; C20: Wood processing and wood, bamboo, rattan, brown, grass products industry; C22: Paper-making and paper products industry; C23: Printing and recording media replication industry; C24: Culture and education, industrial beauty, sports and entertainment products manufacturing industry; C26: Chemical raw materials and chemical products manufacturing industry; C27: Pharmaceutical manufacturing industry; C28: Chemical fiber manufacturing industry; C29: Rubber and plastic products industry; C30: Non-metallic mineral products industry; C33: Metal products industry; C35: Special equipment manufacturing industry; C36: Automobile manufacturing industry; C38: Electrical machinery and equipment manufacturing industry; C39: Computer, communications and other electronic equipment manufacturing industry; C40: Instrument manufacturing industry; C42: Comprehensive utilization industry of waste resources	
	E47, E49–E50	E47: Housing and construction industry; E49: Construction and installation industry; E50: Building decoration and other construction industries	
	F51–F52	F51: Wholesale industry; F52: Retail industry	
	G56	G56: Air transport industry	
	H61–H62	H61: Accommodation industry; H62: Catering industry	
	I64–I65	I64: The Internet and related services; I65: Software and information technology services industry	
	K70	K70: Real estate industry	
	L71–L72	L71: Leasing industry; L72: Business services industry	
	M74–M75	M74: Professional technology services industry; M75: Science and technology promotion and application services	
	R88	R88: Sports

## Footnotes

^1^The principle of “three causes, three preferences, and three disincentives” means “adhere to the principle of applying policies based on local conditions, industries and enterprises, and should be independent, controlled and controlled, and participated in, and should not engage in matchmaking, full coverage and timetable” (Source: Guiding Opinions of the CPC Central Committee and the State Council on Deepening the Reform of State-owned Enterprises, August 24, 2015. http://www.mohrss.gov.cn/xxgk2020/fdzdgknr/zcfg/gfxwj/ldgx/201509/t20150929_221687.html).

^2^The calculation formula is $H_{it}=1-\sum_{i=1}^{n}P_{i}^{2}$ ￼. The *P_i_* represents the proportion of *Group i* members in the total team. The *H* value is between 0 and 1. The higher the *H* value, the higher the heterogeneity of the team.

## Appendix

Industry classification.
